# 

**Published:** 1902-02-22

**Authors:** 


### NOTICE TO CORRESPONDENTS.

All MSS., Letters, Books for Review, and other matters intended for the Editor should be addressed THE EDITOR, "THE HOSPITAL," THE HOSPITAL BUILDING, 28 & 29 SOUTHAMPTON STREET, STRAND, W.C.

The Editor cannot undertake to return rejected MS., even when accompanied by stamped directed envelope.

**Notice to Advertisers and Subscribers.**

All Advertisements, Orders for Copies of THE HOSPITAL, and Business Communications should be addressed to **THE MANAGER** (**Not to the Editor**), THE HOSPITAL, 28 & 29 Southampton Street, Strand, London, W.C.

The Cost of Subscription, post free, is as follows:—Per week, 2½d.; per quarter, 2s. 8d.; per half-year, 5s. 3d.; per annum, 10s. 6d. Foreign:—Per annum, 15s. 2d., payable, in all cases, in advance.

**NOTICE.—Vol. XXX. of "The Hospital" (April 1901, to September 1901), in handsome cloth binding can now be obtained at the office of this Journal, price, 7s. 6d. Cases for binding the Numbers contained in this Volume 1s. 6d. Index to Vol. XXX., 2½d., post free.**
**The Monthly Part for March will be ready on March 1st. Price 6d., by post 8d.**

### SCRAPS AND GLEANINGS.

HIS MAJESTY THE KING has graciously sent a present of 60 lbs. of cast linen for the use of the patients of the Cancer Hospital.

A LADIES' committee has been formed for the purpose of making a house-to-house collection in aid of the Coventry and Warwickshire Hospital. An additional £500 is required to free the hospital from debt, and it is expected that the ladies will be able to collect this amount.

A TENDER of £20,715 odd has just been accepted for the foundations and superstructure of the new Seamen's Hospital to be erected as a memorial of the late Queen's Diamond Jubilee. The committee are anxious to receive all promised subscriptions without delay.

AT the general meeting of the Governors of the West Kent General Hospital the report, which was read by the secretary, stated that for the past year the total number of in-patients was 700, and of out-patients 4,608. There were 1,263 casualties, as against 1,319 the previous year. The Isolation Ward had been occupied during 25 weeks.

At the annual meeting of the subscribers to the Norfolk and Norwich Eye Infirmary, the chairman read the report in which it was stated that the subscription list had been increased by the addition of ten guineas annually from the Earl of Leicester. The report of the medical staff stated that 625 cases had been under treatment during 1901, 125 of these being in-patients.

The largest sum ever received by the Staffordshire General Infirmary was the legacy of £5,000 left to the institution during last year. In addition 14 new subscribers were added to the list. The auditors' report shows that the total expenditure exceeded the total ordinary receipts by £215. The donations amounted to £647 odd.

The honorary secretaries of the Bristol Victoria Jubilee Convalescent Home announce that £10,000 has been presented to the Home to endow 10 free beds, and they have also received £4,115 through the Lord Mayor. The governors will therefore be able to reduce the charge for paying patients from 12s. to 10s. per week. During the past year 1,017 patients have been admitted into the Home.

There is a proposal on foot to establish a Hospital Saturday Fund in Leicester on the lines which have proved so successful in Birmingham, and it is hoped that by this means the new Convalescent Home which is about to be erected will be relieved of any financial difficulty. There is a fund of £18,000 available for the acquisition of the site and erection and equipment of the Home, but a large annual income will be required if the Home is to be a success.

### THE STUDENT OF MEDICINE.

### APPOINTMENTS.

**F. S. Batchelor**, F.R.C.S., appointed Assistant Surgeon to the Dunedin Hospital, New Zealand. **Wm. Donald**, M.D.Aberd., C.M., appointed Certifying Factory Surgeon for the Banff District. **P. G. Garrett**, M.B., B.S.Durh., appointed Certifying Factory Surgeon for the East Shelton District of the County of Leicester. **Jessie D. Granger-Evans**, M.B., Ch.B.Glasg., appointed Clinical Assistant to the New Hospital for Women. **James A. G. Hamilton**, M.B.Dub., L.R.C.S.Edin., appointed Gynæcologist to the Adelaide Hospital. **John B. Hay**, M.B., appointed Medical Officer of Health for the Shire of Bulla, Victoria. **Percy Herbert Liddle**, M.B., Ch.B.Melb., M.R.C.S.Eng., appointed Medical Officer of Health for the Shire of Glenlyon, Victoria. **David H. E. Lines**, M.B., Ch.B.Melb., appointed House Surgeon to the Hobart General Hospital, Tasmania. **J. Allden Lloyd**, M.B.Lond., M.R.C.S., L.R.C.P.Lond., appointed House Surgeon to the Stroud Hospital. **Donald Mackenzie**, M.B., C.M.Aberd., appointed Certifying Factory Surgeon for the Ullapool District of the County of Ross and Cromarty. **J. E. J. Moffit**, L.K.Q.C.P., L.R.C.S.Irel., appointed Government Medical Officer and Public Vaccinator for Balranald. **James S. Ormond**, M.D.Glasg., appointed Medical Officer of Health for the Town of Malvern, Victoria. **J. C. Padwick**, M.R.C.S., L.R.C.P.Lond., appointed Medical Officer of Health for the Bridgnorth Rural District. **W. Wyndham Powell**, F.R.C.S., appointed Surgeon to the Westminster General Dispensary. **Basil A. Spence**, M.B., Ch.B.Edin., appointed Dispensary Surgeon to the Bradford Royal Infirmary. **Gregory Sprott**, M.D.Glasg., D.P.H., appointed Honorary Medical Officer to the Hobart Hospital, Tasmania. **W. Mitchell Stevens**, M.D.Lond., M.R.C.P., appointed Assistant Physician to the Cardiff Infirmary. **Herbert R. Vachell**, M.D.Aberd., appointed Physician to the Cardiff Infirmary. **Hamilton Vaughan**, M.R.C.S., L.R.C.P.Lond., appointed Second Assistant Medical Officer to the St. Marylebone Infirmary, Notting Hill. **J. H. Wilson**, L.R.C.P., L.R.C.S.Edin., appointed Resident Surgeon and Dispenser at Trial Bay Prison, New South Wales. **Thomas F. Wyse**, L.R.C.P., L.R.C.S.Irel., appointed Dispensary Medical Officer for the Philipstown Division of the Tullamore Union.

28 and 29 Southampton Street, Strand, London, W.C.

